# The Emergency Department as an Opportunity for Naloxone Distribution

**DOI:** 10.5811/westjem.2018.8.38829

**Published:** 2018-09-10

**Authors:** Alexander H. Gunn, Zachary P.W. Smothers, Nicole Schramm-Sapyta, Caroline E. Freiermuth, Mark MacEachern, Andrew J. Muzyk

**Affiliations:** * Duke University, Duke-Margolis Center for Health Policy, Durham, North Carolina; † Duke University School of Medicine, Durham, North Carolina; ‡ Duke Institute for Brain Sciences, Department of Psychiatry, Duke University, Durham, North Carolina; § Duke University School of Medicine, Division of Emergency Medicine, Durham, North Carolina; ¶ Taubman Health Sciences Library, University of Michigan, Ann Arbor, Michigan; || Duke University Hospital, Department of Pharmacy, Durham, North Carolina; # Campbell University College of Pharmacy and Health Sciences, Department of Pharmacy Practice, Buies Creek, North Carolina

## Abstract

**Introduction:**

Substance use disorders, including opioid use disorders, are a major public health concern in the United States. Between 2005 and 2014, the rate of opioid-related emergency department (ED) visits nearly doubled, from 89.1 per 100,000 persons in 2005 to 177.7 per 100,000 persons in 2014. Thus, the ED presents a distinctive opportunity for harm-reduction strategies such as distribution of naloxone to patients who are at risk for an opioid overdose.

**Methods:**

We conducted a systematic review of all existing literature related to naloxone distribution from the ED. We included only those articles published in peer-reviewed journals that described results relating to naloxone distribution from the ED.

**Results:**

Of the 2,286 articles we identified from the search, five met the inclusion criteria and had direct relevance to naloxone distribution from the ED setting. Across the studies, we found variation in the methods of implementation and evaluation of take-home naloxone programs in the ED. In the three studies that attempted patient follow-up, success was low, limiting the evidence for the programs’ effectiveness. Overall, in the included studies there is evidence that distributing take-home naloxone from the ED has the potential for harm reduction; however, the uptake of the practice remained low. Barriers to implementation included time allocated for training hospital staff and the burden on workflow.

**Conclusion:**

This systematic review of the best evidence available supports the ED as a potential setting for naloxone distribution for overdose reversal in the community. The variability of the implementation methods across the studies highlights the need for future research to determine the most effective practices.

## INTRODUCTION

In April 2018, the United States (U.S.) Office of the Surgeon General released a public health advisory urging communities to improve access to naloxone for those who are at risk for opioid overdose.^1^ This recommendation is shared in the 2017 President’s Commission on Combating Drug Addiction and the Opioid Crisis, and the World Health Organization’s guidelines that recommend increased access to naloxone.^2^,^3^ These recommendations are supported by previous research, which demonstrated that community-based, take-home naloxone distribution is associated with reduced opioid-overdose death rates and is cost effective.^4^–^6^ A national survey of community-based naloxone distribution programs found that from 1996 to 2014 152,284 individuals received naloxone from a community-based program, which resulted in the successful reversal of 26,463 overdoses.^4^ Despite the high number of reversals, take-home naloxone programs are only present in 8% of U.S. counties overall and 12% of counties with the highest opioid-overdose rate.^7^ To improve access to take-home naloxone, community distribution programs have expanded to include substance use treatment facilities, primary care clinics, and pharmacies.^4^ The emergency department (ED) presents another opportunity to further expand access to take-home naloxone.

Over the last decade, the number of opioid-related ED visits has dramatically increased. From 2005 to 2014, these visits nearly doubled from 89.1 to 177.7 per 100,000 people, and more recent Centers for Disease Control and Prevention (CDC) estimates indicate an even sharper increase has occurred since 2015.^8^,^9^ This rise in ED visits positions the ED as a powerful venue for identification of patients with substance use disorder (SUD) needs that, if unmet will result in higher hospital and ED admissions and healthcare costs.^10^ This large pool of patients also provides an opportunity for healthcare workers to engage patients with opioid use disorder (OUD) and provide evidence-based interventions such as take-home naloxone.

Naloxone, a U.S. Food and Drug Administration-approved opioid overdose antidote, is a proven viable, safe, and effective intervention that can reduce opioid-overdose deaths in the community setting and be effectively administered by lay people. It has decreased ED visits when co-prescribed with opioid medications.^1^,^5^,^11^,^12^ Pulmonary edema has been reported following the administration of naloxone; however, the best evidence has indicated these cases are multi-factorial and that naloxone is recommended in the case of opioid overdose.^13^,^14^

Previous research has demonstrated that an OUD intervention in the ED can reduce overdose risk and that ED providers are willing to prescribe take-home naloxone; however, they have low confidence in doing so.^15^,^16^ Further, the majority of patients at risk for opioid overdose in the ED are willing to accept a take-home naloxone kit and believe that the ED is an appropriate venue.^17^ Healthcare workers in the ED who want to implement a take-home naloxone program must be able to refer to the literature to understand the available evidence. The purpose of this systematic review was to identify, evaluate, and summarize available evidence regarding the distribution of take-home naloxone in the ED and identify the areas that require future research.

## METHODS

This review adheres to the Preferred Reporting Items for Systemic Reviews and Meta-analyses (PRISMA) guidelines.^18^ We did not conduct a meta-analysis due to the heterogeneity of study interventions, assessments, and analysis of collected data. Extracting and grading the evidence was not possible due to the variation in outcome measures and design across included studies.

### Literature Search

One author (MM) performed comprehensive searches in Ovid MEDLINE, Ovid MEDLINE In-Process & Other Non-Indexed Citations, Ovid MEDLINE Epub Ahead of Print, Embase.com, Cochrane Central Register of Controlled Trials, and CINAHL via the EBSCOhost research platform. The searches were initially run in June 2017 and rerun for the final time in April 2018. Each search consisted of a combination of ED and naloxone terminology, with appropriate, controlled vocabulary and title and abstract keyword variations. The searches yielded 2,286 citations after duplicates were removed in Endnote X6 (Clarivate Analytics). We excluded conference abstracts and conference papers from the Embase search. The searches were otherwise free of restrictions. The Ovid MEDLINE search is included in Table 1 and all complete, reproducible searches are available in a data repository at doi:10.7302/Z2WD3XSM.

### Eligibility Screening

Two authors (AG and ZS) independently reviewed the titles and abstracts of all retrieved and included articles that described naloxone distribution from the ED. A third author (AM) resolved any disagreements to remove selection and scoring bias. All included papers were reviewed for any additional articles not identified in the literature database search.

The inclusion criteria required that articles do the following: (1) Be or include original research with outcomes; (2) describe a naloxone distribution from the ED; and (3) create an intervention targeted to individuals with OUD, SUD, or current opioid use. We excluded conference proceedings, thesis papers, white papers, policy recommendations, and abstracts. Although the literature search was not limited to English-only articles, all records identified through database searches were published in English. Of the records screened, the most common reasons for exclusion were not describing naloxone distribution initiatives, not describing distribution from the ED specifically, and inappropriate publication types such as dissertations or poster abstracts. Five articles met all of the inclusion criteria as shown in the PRISMA flow diagram in Figure.

## RESULTS

Five articles out of the 2,286 we identified met the inclusion criteria and had direct relevance to the naloxone distribution from the ED setting. The included articles varied in study design from randomized clinical trial (1) to prospective cohort studies (2), retrospective qualitative analysis (1), and descriptive study (1).

Across the studies, there is variation in the methods of implementation and evaluation of ED take-home naloxone programs. These methods of implementation included grant-funded counselors available to perform the intervention, medical student volunteers to screen patients in the ED, electronic health record (EHR) alerts that notified providers of eligible patients, and a physician’s assistant (PA) with training in addiction medicine. The methods of evaluation included two studies that examined the rate of prescribing take-home naloxone, two that followed up with patients to determine effectiveness of the intervention, and one that examined the amount of time between the intervention and the next EHR-recorded opioid overdose.

In the three studies that attempted patient follow-up, the rate of successful follow-up was low, which limits the evidence for effectiveness. Authors attributed the poor follow-up to social and economic factors of the patient population, including that a majority of enrolled patients were homeless or living in impermanent housing. In the included studies, there is evidence that distributing take-home naloxone from the ED has the potential of harm reduction; however, the uptake of the practice remained low. Barriers to implementation included time allocated for training hospital staff and the burden that distribution and counseling place on ED workflow.

### Banta-Green et al.^19^

This randomized clinical trial identified 241 adults at risk for opioid overdose in two hospital EDs and placed participants to either overdose education with a brief behavioral intervention and take-home naloxone, or usual care. Participants were identified through EHR review or staff referral and the majority of participants were male, white, non-Hispanic, homeless, unemployed, and more than half had used opioids every day of the previous month. The 30-minute intervention was conducted by interventionists with a master’s degree who had basic training in motivational interviewing.

The primary outcome was the number of opioid-related events recorded in the EHR following the intervention for the intervention and control group. The authors found no significant difference in the number of opioid events between the control and intervention group as well as no significant difference in the time to the first overdose between the groups. The authors concluded that the null findings may have been the result of the low housing security in their study population and that more intensive interventions may have been necessary to have substantial impact on opioid overdoses. The study did not report self-reported overdoses or the use of naloxone administration due to low follow-up rates. Finally, the authors suggested that due to the constraints of timing and space in the ED, a more concise overdose and naloxone training may be sufficient and congruent with the population-level benefit in mortality rates in communities with greater rates of naloxone distribution.

### Barbour et al. 20

This prospective cohort study included 24 patients at risk of opioid overdose. In the ED, two medical students trained in harm reduction identified patients with an opioid- or overdose-related chief complaint. Participants completed a brief survey, and the medical students then delivered education in overdose reversal and naloxone usage, which took approximately 15 minutes per participant. The treating physician prescribed naloxone to eligible patients, which could be filled after discharge.

While 71 patients at risk of opioid overdose presented to the ED during this study and 43 were interested in the study, only 24 were included. For 16 eligible participants, the treating physician refused to prescribe naloxone and as a result they were excluded. Seven of the 24 patients enrolled in the study were successfully contacted for the three-month follow-up. Of these seven patients, only two had filled their prescription despite none of the other participants reporting obstacles to obtaining naloxone. The authors concluded that the greatest barrier to take-home naloxone in the ED was physician resistance. The authors believed that the high number of patients whose physician would not prescribe naloxone emphasizes the need to improve physician education about harm reduction. Another identified barrier was the pharmacy policy that prevented the ED from providing take-home naloxone directly at discharge.

### Devries et al. 21

This descriptive study of a healthcare systemwide quality improvement project describes a multisite, interdepartmental effort to increase take-home naloxone access for patients at risk for opioid overdose. This widespread initiative included the development of prescribing guidelines, educational materials for providers, EHR alerts and order sets, and the inclusion of all types of naloxone in standard pharmacy stock. In the ED, a medical student screened patients for opioid-overdose risk and eligibility for take-home naloxone. Once identified, providers would prescribe take-home naloxone and had the option of billing private insurance when available or the use of internal funds to cover the cost of naloxone for patients that were un- or under-insured.

Across the health system, the education program conducted 13 training sessions in eight departments. In the ED, specifically, 40 of the 98 physicians and 40 of the 184 nurses completed the training. In 2015, the ED had zero prescriptions for take-home naloxone and from May 2016 to September 2016, they prescribed 46 take-home naloxone kits. Of all the naloxone prescriptions, 43% were intramuscular, 53% were intranasal, and 4% were naloxone auto-injectors. The EHR alert led to a prescription for take-home naloxone 14% of the time. The authors emphasized the need for more-targeted EHR alerts to increase the rate of prescriptions and avoid alert fatigue. The study results showed that take-home naloxone programs can be initiated at large, multisite health systems and, specifically, within the ED.

### Drainoni et al.^22^

This study retrospectively examined the uptake of nasal naloxone distribution in the ED following the implementation of a new policy encouraging the intervention. The study team supplemented this data with qualitative interviews of the ED staff. In the eight months prior to policy implementation, 8% of ED patients at risk for opioid overdose received take-home naloxone kits. The low distribution rate was attributed to a variety of factors, including lack of knowledge of the intervention. In addition to broader distribution of naloxone, the new policy meant that take-home naloxone kits were available 24 hours a day. Despite this, in the eight months following the policy initiation, only 7% of ED patients with the same overdose risks received take-home naloxone in the ED. Despite the low uptake, the qualitative interviews with ED staff revealed strong philosophical acceptance of the intervention. The barriers to implementation identified from interviews included logistical workflows, ambiguous staff roles, and lack of education.

The authors concluded that the successful implementation of a naloxone distribution in the ED setting is largely driven by factors other than acceptance by providers. The specific recommendations for establishing implementation included the following: creating a focused target population with a high degree of risk to initiate the innovation; developing training to engage providers in overdose prevention and harm reduction; and identifying at least one clinical champion from each role in the ED.

### Dwyer et al.^23^

This prospective cohort study included 415 patients who were at risk for opioid overdose. A PA approached those patients to provide education about overdose risks as well as how to recognize and respond to an overdose. Of this group, 359 received opioid education only and 56 received opioid education and naloxone. The delivered opioid education and naloxone distribution took five minutes. Each kit cost 55 dollars for two atomized 2 mg naloxone vials; these were funded by the Massachusetts Department of Public Health. One year following the ED visit, these patients were contacted for a telephone survey.

Fifty-one of the original group of patients completed the survey: 37 patients who had received opioid education and naloxone, and 14 who received opioid education only. Of those who completed the survey, over half (53%) had witnessed an overdose since their ED visit. Moreover, within the group that witnessed an overdose, the majority (65%) called 911 and nearly all (93%) stayed with the victim. Of those who received a naloxone kit within the surveyed group, 16% reported using their kit to successfully reverse a witnessed overdose, which is consistent with previous reports of take-home naloxone programs distributed in the community.^4^

The study authors concluded that the ED is a promising opportunity for opioid overdose harm reduction and naloxone distribution to laypersons. While the results of the study demonstrated the potential for the ED setting, this study was limited by its low follow-up interview enrollment. Only 12% of the patients who received either intervention completed the survey; however, over 50% of the group that received naloxone participated in the survey.

### Implementation Considerations

The variability of the implementation methods across the studies highlights the need for future research to determine the most effective practices. The following categories are general themes for implementation considerations: (1) Identification of personnel; (2) education for providers and staff, (3) EHR integration; (4) patient identification methods; (5) funding for take-home naloxone; and (6) method of dispensing take-home naloxone. Table 2 contains detailed explanations for these implementation considerations.

## DISCUSSION

On the basis of the evidence available, the ED represents a potential opportunity to engage patients at risk for overdose and distribute take-home naloxone for overdose reversal in the community. The reviewed work demonstrates that patients at risk of opioid overdose presenting to the ED are willing to accept take-home naloxone, which is consistent with previous related research.^17^,^19^,^20^,^23^ While the evidence regarding the effectiveness of the intervention is poor, one study reported that16% of patients who received naloxone kits went on to use it in the rescue of an opioid overdose.^23^ Even with this potential for harm reduction and the acceptance among patients and providers, the practice of prescribing take-home naloxone was overall low.^20^–^23^

In addition to identifying the ED as an opportune setting to distribute naloxone, the included studies provide insight on the potential barriers and enabling factors for implementation as shown in Table 2. These considerations are continuing to change as the environment around naloxone distribution is developing. Many states have expanded naloxone-access laws, allowing a provider to write a standing order for an entire group of people, such as medical students, for example, to distribute naloxone kits. Additionally, private insurance companies are publicly making intranasal naloxone available with very little or no co-pay. The majority of the included studies as well as previous research has shown that providers are accepting of take-home naloxone programs and willing to prescribe.^15^,^19^,^21^–^23^ In one study, however, physician resistance to prescribing naloxone was identified as the key barrier.^20^ The reasons for the experienced resistance are unclear and emphasize the importance of developing training to engage providers before initiating the intervention and identifying a program champion.

The included studies have low rates of patient follow-up, which limits our understanding of the effectiveness of take-home naloxone from the ED. The absence of this evidence may deter other EDs from attempting to implement such a program. This course of action would not be consistent with the recommendations of the authors in each of the included articles and the previous research that has shown community-based naloxone distributions are cost effective and decrease mortality.^5^,^6^,^19^–^23^ While more research is needed to determine the best methods and to measure effectiveness of ED programs, the low rate of follow-up is likely the result of this difficult-to-track population, which is largely homeless and unemployed.^19^ The ED can reach patients at risk for overdose who do not present to other healthcare venues. Thus, the potential for harm reduction signals the power of further engagement of patients at risk for overdose in the ED.

This review is the first to analyze previous research related to take-home naloxone distribution from the ED. While there are few studies published, the results show that such programs are feasible and could be an effective venue for harm-reduction strategies in the face of the rising number of opioid-related ED visits. Clinicians and hospital leadership should consider strategies to promote the distribution of naloxone to at-risk patients from the ED. Future work that examines the relative effectiveness of distributing take-home naloxone, motivational counseling, and connecting patients with evidence-based treatment could be vital in creating effective methods. Additionally, more research is needed to improve the real-time identification of at-risk patients and to understand which formulation of naloxone is most effective for take-home use.

## LIMITATIONS

Only five articles met the inclusion criteria. This small sample size highlights the need for future research but also provides little evidence to support claims. The inclusion criteria only allowed for peer-reviewed, published literature to be reviewed. The authors recognize that ED-based, take-home naloxone programs may exist around the country but have not been reported on. Further, literature that described naloxone distribution from settings other than the ED was excluded, which limited the possibility of expanding findings to outside the ED. Finally, we could not conduct a meta-analysis due to the low number of included studies and heterogeneity of outcomes, which limits the conclusions that can be drawn from this review.

## CONCLUSION

The systematic review of the best evidence available supports that the ED is a potential setting to distribute naloxone for overdose reversal in the community. The variability of the implementation methods across the studies highlights the need for future research to determine the most effective practices.

## Figures and Tables

**Figure:**
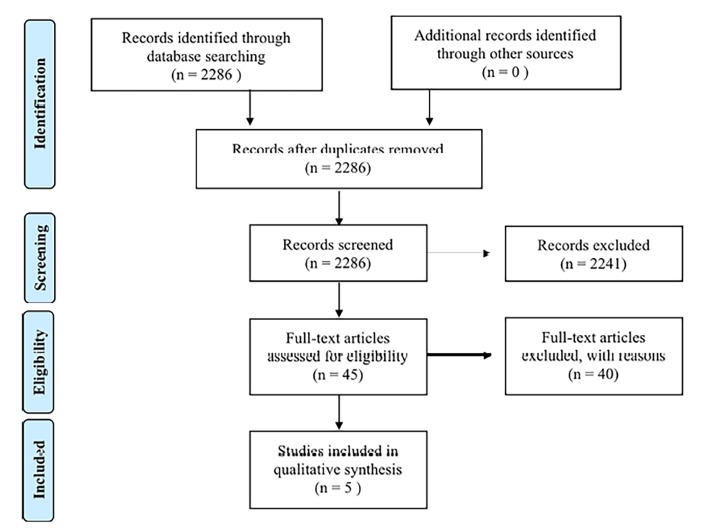
Literature search and article selection.

**Table 1:** Literature search strategies regarding naloxone access for the three Ovid MEDLINE databases.

Search #	S1earch statement
1	exp naloxone/ or (antioplaz or curamed or maloxone or mapin or nalone* or naloxon* or narcan or narcanti or narcon or ratiopharm or zynox).tw. or (opioid* or opiate*).ti.
2	exp emergency medical services/ or exp emergency treatment/ or emergenc*.ti. or (emergenc* adj2 (depart* or room* or service* or unit* or ward or wards)).tw.
3	and/1–2

**Table 2:** Implementation considerations for take-home naloxone programs in the emergency department.

Identification of personnel	Included studies used health counselors, medical student volunteers, PAs, pharmacists, physicians, and nurses.^19^–^23^
Education and training	Lack of time available for workforce training was identified as a key barrier to successful implementation.^22^
EHR integration	Only 14% of EHR notifications resulted in a prescription for take-home naloxone. Authors identified that more targeted alerts could be more effective.^21^
Patient identification and workflow	The identification of patients in the included studies was done through provider referral, listed chief complaint, listed diagnosis, and screening questionnaires.^19^–^23^ One study recommended starting with a specific high-risk population in the ED to implement the practice and scale to other at-risk patient populations.^22^
Source of funding for take-home naloxone kits	Take-home naloxone kits were funded in a variety of methods, including grant funding, billing private insurance, billing Medicaid or Medicare, and relying on a cross-sector partnerships with local and state health departments.^19^–^23^
Pharmacy considerations	In two studies, even when naloxone was prescribed, very few were filled. To this end, a common factor identified as an enabling factor was ED patients being able to leave the ED with the take-home naloxone kits at any time of day.^20^,^22^ Further, the type of naloxone distributed across the studies varied. The most common was a mucosal atomizer kit with a vial of naloxone.^19^–^23^

*EHR,* electronic health records; *ED*, emergency department.
